# Strengthening pathogen genomic surveillance for health emergencies: insights from the World Health Organization’s regional initiatives

**DOI:** 10.3389/fpubh.2023.1146730

**Published:** 2023-06-09

**Authors:** Oluwatosin Wuraola Akande, Lisa L. Carter, Abdinasir Abubakar, Rachel Achilla, Amal Barakat, Nicksy Gumede, Alina Guseinova, Francis Yesurajan Inbanathan, Masaya Kato, Etien Koua, Juliana Leite, Marco Marklewitz, Jairo Mendez-Rico, Chavely Monamele, Biran Musul, Karen Nahapetyan, Dhamari Naidoo, Rachel Ochola, Mehmet Ozel, Philomena Raftery, Andrea Vicari, Pushpa Ranjan Wijesinghe, Joanna Zwetyenga, Kelly Safreed-Harmon, Céline Barnadas, Mick Mulders, Dmitriy I. Pereyaslov, Jilian A. Sacks, Taylor Warren, Sébastien Cognat, Sylvie Briand, Gina Samaan

**Affiliations:** ^1^Epidemic and Pandemic Preparedness and Prevention, World Health Organization, Geneva, Switzerland; ^2^Country Readiness Strengthening, World Health Organization Lyon Office, Lyon, France; ^3^Infectious Hazard Prevention and Preparedness, World Health Organization Regional Office for the Eastern Mediterranean, Cairo, Egypt; ^4^Emergency Preparedness and Response, World Health Organization Regional Office for Africa, Brazzaville, Democratic Republic of Congo; ^5^Infectious Hazard Management, World Health Organization Regional Office for Europe, Copenhagen, Denmark; ^6^WHO Health Emergencies, Regional Office for South-East Asia, New Delhi, India; ^7^PAHO Health Emergencies, Pan American Health Organization, Washington DC, United States; ^8^WHO Health Emergencies Programme, World Health Organization Country Office, Ankara, Türkiye; ^9^Independent Consultant, Barcelona, Spain; ^10^Immunization, Vaccines and Biologicals, World Health Organization, Geneva, Switzerland; ^11^Disaster Risk Management and Resilience, World Health Organization, Geneva, Switzerland

**Keywords:** pathogen genomic surveillance, pathogen sequencing, pathogen genomics, molecular epidemiology, public health surveillance, public health laboratories, health emergencies, COVID-19

## Abstract

The onset of the COVID-19 pandemic triggered a rapid scale-up in the use of genomic surveillance as a pandemic preparedness and response tool. As a result, the number of countries with in-country SARS-CoV-2 genomic sequencing capability increased by 40% from February 2021 to July 2022. The Global Genomic Surveillance Strategy for Pathogens with Pandemic and Epidemic Potential 2022–2032 was launched by the World Health Organization (WHO) in March 2022 to bring greater coherence to ongoing work to strengthen genomic surveillance. This paper describes how WHO’s tailored regional approaches contribute to expanding and further institutionalizing the use of genomic surveillance to guide pandemic preparedness and response measures as part of a harmonized global undertaking. Challenges to achieving this vision include difficulties obtaining sequencing equipment and supplies, shortages of skilled staff, and obstacles to maximizing the utility of genomic data to inform risk assessment and public health action. WHO is helping to overcome these challenges in collaboration with partners. Through its global headquarters, six regional offices, and 153 country offices, WHO is providing support for country-driven efforts to strengthen genomic surveillance in its 194 Member States, with activities reflecting regional specificities. WHO’s regional offices serve as platforms for those countries in their respective regions to share resources and knowledge, engage stakeholders in ways that reflect national and regional priorities, and develop regionally aligned approaches to implementing and sustaining genomic surveillance within public health systems.

## Introduction

Prior to the COVID-19 pandemic, disease control programmes such as influenza, measles, poliomyelitis, tuberculosis, and antimicrobial resistance were using sequencing for pathogen characterization and monitoring, as well as for the development of vaccines, therapeutics and diagnostics. However, the COVID-19 pandemic called much greater attention to the role of genomic surveillance as an essential public health tool.

**Figure 1:**
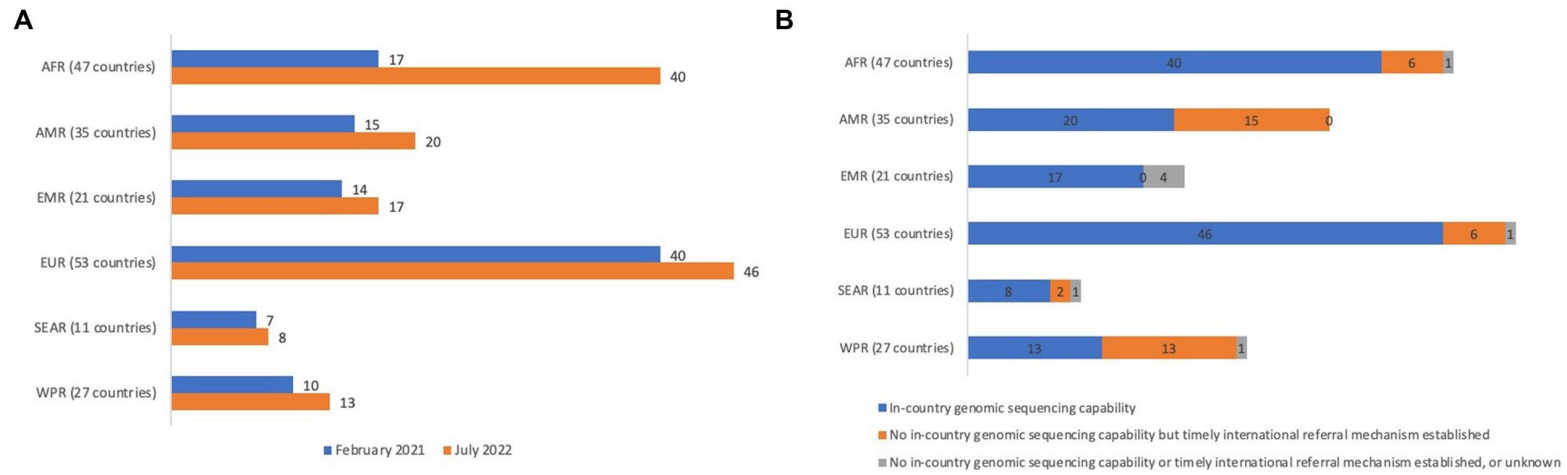
**(A)** Increases in country capability to perform in-country genomic sequencing of SARS-CoV-2, by region of the World Health Organization, February 2021 – July 2022 (*N* = 194). **(B)**. Country capability to perform in-country genomic sequencing or utilize timely* international referral mechanisms for pathogens with pandemic and epidemic potential, by region of the World Health Organization, July 2022 (*N* = 194). AFR = African Region, AMR = Region of the Americas, EMR = Eastern Mediterranean Region, EUR = European Region, SEAR = South-East Asia Region, WPR = Western Pacific Region. * Timely is defined as triggering genomic sequencing within 7 days of event or pathogen detection. Source: World Health Organization, as of July 2022. “Countries” are World Health Organization Member States.

Given the potential for pandemics and epidemics to quickly spread across borders, information about the pathogen genome that is driving a health emergency in one country can greatly aid preparedness and response measures at the national, regional and global levels. In response to COVID-19, many countries rapidly established SARS-CoV-2 sequencing capacities or leveraged capacities used for other disease control programmes. The number of countries with in-country SARS-CoV-2 genetic sequencing capability increased by 40% from February 2021 to July 2022 (Figure 1A). Furthermore, 84% of the 50 countries that lacked in-country sequencing capability as of July 2022 reported having access to timely international referral mechanisms (Figure 1B). In the wake of these efforts, genomic surveillance is now widely viewed as a core component of pandemic preparedness and response systems globally (1–3).

Sustaining and further expanding national genomic sequencing capability will require countries and the global community at large to address many challenges. Depending on the country context, these challenges may include the high cost of sequencing; unreliable supply chains for equipment and consumables; insufficient workforce, particularly for bioinformatics; weak systems for integrating genomic data with epidemiological and clinical data; constraints on sharing data; and concerns about the implications of data-sharing (4). These challenges can lead to suboptimal use of genomic surveillance to inform public health action. They might also hinder the rapid scale-up of systems during future pandemics or epidemics.

Recognizing the need for coherence in addressing challenges in the genomic surveillance landscape at all levels, WHO launched the Global Genomic Surveillance Strategy for Pathogens with Pandemic and Epidemic Potential 2022–2032 (the “Global Strategy”) in March 2022. The Global Strategy’s key measure of success is that: *By 2032, all 194 WHO Member States* [hereafter, “countries”] *have, or have access to, timely genomic sequencing for pathogens with pandemic and epidemic potential* (4). A wide array of stakeholders from the human, animal and environmental health sectors are contributing to achieving this target including governments; intergovernmental agencies; global and regional networks; technical partners; donors; academic institutions; and the private sector.

As the steward of the Global Strategy, WHO provides global leadership, coordination, advocacy, and technical guidance to facilitate the strategy’s implementation. WHO convened the Partners Coordination Group (PCG) to maximize coherence and identify opportunities for collaboration among genomic surveillance stakeholders in relation to the strategy’s objectives (5). The PCG’s 30 member organizations, which come from all six WHO regions, include multilateral agencies, funding bodies, nongovernmental organizations, and academic institutions. WHO’s global programme of work includes the International Pathogen Surveillance Network (6), BioHub (7), and pathogen-specific initiatives (8, 9). These are all platforms for addressing challenges and sustainably extending access to timely genomic sequencing of pathogens with pandemic and epidemic potential to all countries worldwide.

WHO works through its six regional offices and 153 country offices to support country-owned efforts to strengthen genomic surveillance in all 194 countries (Supplementary Figure 1). To effectively implement the Global Strategy, there is a need to address knowledge gaps in terms of existing systems and capacities, challenges and enablers in the genomic surveillance architecture.

The purpose of this article is to share insights from WHO’s regionally contextualized work to facilitate the implementation of the Global Strategy, including challenges and enablers at the regional level. The paper also aims to identify pathways to ensure the sustainability of genomic surveillance for global pandemic preparedness and response. These insights emerged from semi-structured interviews conducted among co-authors who are WHO staff working on implementing pathogen genomic surveillance for pathogens with pandemic and epidemic potential at the global and regional levels.

## Regional approaches to strengthening genomic surveillance capacities

Contextual factors such as those relating to epidemiological dynamics, health system capacities, and country priorities have given rise to different regional approaches to supporting implementation of the Global Strategy, as described in the following sections. Table 1 summarizes contextual challenges and enablers for implementation in the six WHO regions.

**Table 1:** Regional contexts, laboratory capacities, challenges, and enablers for strengthening genomic surveillance capacities.

Region	Context, laboratory capacities and challenges^1^	Enablers
African Region (47 countries)	Financial, logistical and laboratory system obstacles to procuring and shipping equipment and supplies, as well as to dispatching genomic sequencing samples, particularly in fragile states or those experiencing embargoes	Country commitment to developing and implementing national strategies or roadmaps to strengthen capacity in national public health institutes
	Insufficient public sector health workforce with core skills required for performing laboratory, bioinformatics and molecular epidemiological analysis	Robust regional and global partner support to countries to strengthen laboratory and surveillance systems
	Countries in the region experience frequent outbreaks of high-threat pathogens including viral hemorrhagic fevers such as Ebola, Sudan virus disease, and Lassa fever, destabilizing laboratory strengthening efforts	
	Laboratory core capacity scores in reporting countries range from 28 to 92 (regional average: 61)	
Region of the Americas (35 countries)	Almost half (46%) of countries are small island developing states^2^	Established a collaborative sequencing network bringing together designated regional reference sequencing laboratories, countries with in-country genomic sequencing, and countries that ship samples to regional reference sequencing laboratories
	Laboratory costs are high, with limited access to supplies and equipment servicing	Partnerships between public and private institutions to strengthen genomic surveillance by improving access to tools and workforce development in the region
	Regional high-threat pathogens include respiratory viruses and arboviruses	
	Laboratory core capacity scores in reporting countries range from 44 to 100 (regional average: 75)	
Eastern Mediterranean Region (21 countries)	Financial, logistical and laboratory system obstacles to procuring and shipping equipment and supplies, as well as to dispatching genomic sequencing samples, particularly in countries with complex emergencies or those experiencing embargoes	Rapid expansion of established SARS-CoV-2 sequencing capacities to other priority pathogens
	Difficulty retaining skilled public health workers who have opportunities to transition to better-paying jobs in-country and abroad	Ongoing capacity building and training activities for sequencing and bioinformatics
	Regional high-threat pathogens include Crimean Congo hemorrhagic fever virus, influenza virus, *Vibrio cholerae*, Middle East respiratory syndrome virus and Rift Valley fever virus	Three regional laboratories to support countries in real time
	Laboratory core capacity scores in reporting countries range from 24 to 100 (regional average: 72)	
European Region (53 countries)	Insufficient awareness among partners and donors of the needs of countries that have low capacities	Long-established sequencing capacity in many countries
	Regional high-threat pathogens include respiratory viruses such as influenza, Crimean-Congo hemorrhagic fever, West Nile fever, anthrax and brucellosis	Country-led efforts to develop and implement national genomic surveillance strategies
	Laboratory core capacity scores in reporting countries range from 44 to 100 (regional average: 83)	Collaboration between public and private institutions to build genomic surveillance capacities, particularly in countries with limited resources
South-East Asia Region (11 countries)	Large population (about a quarter of global population) with a need to decentralize capacities in highly populous countries	Regional consortium approach to build and sustain genomic surveillance capacities
	Insufficient public sector health workforce with core skills required for genomic surveillance	Partnerships with in-country and international research institutions to provide technical expertise and resources for genomic surveillance
	Regional high-threat pathogens include those at the human-animal interface such as Nipah virus, respiratory viruses such as influenza, foodborne pathogens and arboviruses such as dengue	
	Laboratory core capacity scores in reporting countries range from 48 to 84 (regional average: 73)	
Western Pacific Region (27 countries)	More than half of countries are small island developing states^2^	Clear regional seven-step approach to build and sustain genomic surveillance, leveraging existing capacities
	Laboratory costs in remote locations are high, with limited access to supplies, connectivity, equipment servicing and referral laboratories	Commitment to strengthening workforce for sequencing and bioinformatics
	Obstacles to streamlining and integrating genomic surveillance data into timely multisource health information systems	
	Regional high-threat pathogens include leptospirosis, typhoid fever, cholera, respiratory viruses such as influenza and arboviruses such as dengue	
	Laboratory core capacity scores in reporting countries range from 56 to 100 (regional average: 80)	
	Laboratory costs in remote locations are high, with limited access to supplies, connectivity, equipment servicing and referral laboratories	

1 Under the International Health Regulations (IHR, 2005), laboratory core capacities are needed to detect, assess and respond promptly and effectively to public health risks. This covers systems for specimen referral, laboratory quality, biosafety and biosecurity, testing capacity modalities, and diagnostic networks. Each year, countries self-score and report on their core capacities. Data shown here summarize laboratory core capacity scores reported for 2022 from 182 of 196 State Parties, with the global average score 74 (range 24–100). [Source: World Health Organization, “e-SPAR State Party Annual Report,” available at: https://extranet.who.int/e-spar (Accessed April 18, 2022)].

2 World Health Organization. WHO country presence in small island developing states (SIDS). (2021). Available at: https://www.who.int/publications/i/item/9789240027657 [Accessed December 20, 2022].

### African region

At the onset of the COVID-19 pandemic, the African region faced the challenge of having low overall laboratory and genomic sequencing capability in a geographically large area with many countries (10). WHO’s Regional Office and the Africa Centres for Disease Control and Prevention (Africa CDC) jointly established the Sequencing Laboratory Network for COVID-19 and Other Emerging Pathogens in September 2020 (11). This work leveraged existing initiatives including capacities of the Africa CDC Pathogen Genomics Initiative, which was launched in 2020. The network first prioritized building genomic sequencing capability in the countries of southern Africa early in the pandemic, when COVID-19 disease incidence was heavily concentrated in that subregion, then expanded to support the entire African Region. In 2021, WHO’s Regional Office decentralized its genomic surveillance activities to work more closely with countries. As part of this approach, the Regional Office established three subregional hubs including the South Africa-based Regional Centre of Excellence for Genomic Surveillance and Bioinformatics, which operates in partnership with the South African National Bioinformatics Institute to support 14 countries (12). The Transforming African Surveillance Systems flagship programme, introduced by the WHO Regional Office for Africa in 2022, brings political attention to and advocates for the use of genomic surveillance in the region within the context of strengthening broader surveillance systems for emergency preparedness and response (13).

### Region of the Americas

The Pan American Health Organization, which serves as WHO’s Regional Office for the Americas, launched the Regional SARS-CoV-2 Genomic Surveillance Network (COVIGEN) in March 2020 to support the generation and timely sharing of SARS-CoV-2 genomic sequencing data in the Region of the Americas. COVIGEN brings together three types of laboratories: those that perform in-country genomic sequencing, those that send samples to regional reference laboratories, and those that serve as regional reference laboratories. COVIGEN currently includes 33 laboratories in 30 countries. Through COVIGEN, the Pan American Health Organization has collaborated with reference laboratories to provide standardized protocols, generate sample selection criteria, and assist with procurement, training and analysis. For countries that lack in-country genomic sequencing capability, the Pan American Health Organization has facilitated the selection and shipping of samples for analysis by reference laboratories. The region’s 35 countries approved the Strategy on Regional Genomic Surveillance for Epidemic and Pandemic Preparedness and Response at the Pan American Sanitary Conference in September 2022 (14). The strategy encourages countries to build on genomic surveillance capabilities that were established in response to SARS-CoV-2 in order to address other emerging or re-emerging pathogens. It also calls for collaboration across existing disease-specific genomic surveillance networks.

### Eastern Mediterranean region

In collaboration with national, regional and global partners, WHO is coordinating the establishment of the Eastern Mediterranean Region Genomic Surveillance Network for Emerging and Re-emerging Infectious Diseases with Pandemic Potential. As of this writing, more than one-third of the Eastern Mediterranean Region’s countries were experiencing crises that WHO categorized as emergencies (15), and many faced major barriers to performing in-country genomic sequencing or shipping samples to the region’s three reference laboratories. WHO’s Regional Office has therefore tailored its genomic surveillance activities taking into account the high incidence of emergencies. One of three regional strategic priorities is to establish and sustain genomic surveillance for countries with complex emergencies (16). Despite the technical challenges associated with bringing sequencing capability to countries with complex emergencies, this approach was deemed more viable than relying on referral networks to ensure timely genomic sequencing for pathogens with pandemic and epidemic potential. Other priorities are building national genomic sequencing capability in non-emergency countries and establishing regional genomic sequencing reference laboratories. While SARS-CoV-2 has been a major focus of genomic surveillance efforts, cholera, Crimean-Congo hemorrhagic fever and Middle East respiratory syndrome also have been sequenced to guide responses to outbreaks of these diseases. The Regional Office for the Eastern Mediterranean Region plans to encourage the broad application of genomic surveillance capability to address the region’s diverse infectious disease threats as they emerge.

### European region

The WHO Regional Office for Europe has worked through multiple pathways to strengthen genomic surveillance capability in the WHO European Region. Genomic surveillance is addressed in the workplan for the region’s High-Threat Pathogens Lab Task Force (17) and is a major focus of the regional laboratory workplan. A strong regional reference laboratory network provides sequencing services and technical support to countries. Additionally, the WHO Regional Office for Europe convenes a bimonthly “COVID-19 variants and mutations updates call” for the region’s ministries of health to share and discuss genomic surveillance data. A subregional focus has been supporting the development of a genomic sequencing hub at the Institute of Public Health of North Macedonia in collaboration with the United Kingdom Health Security Agency’s New Variant Assessment Platform. The WHO Regional Office for Europe in collaboration with the WHO Country Office in Türkiye has been supporting countries to develop national genomic surveillance strategies through a series of consultations in Caucasus and central Asian countries. In addition, a training programme on next-generation sequencing, bioinformatics and molecular epidemiology, developed by WHO Türkiye in consultation with experts from all six WHO regions, is being rolled out across the region.

### South-East Asia region

With 11 countries containing one-quarter of the world’s population, WHO’s Regional Office for South-East Asia has focused on short-term genomic surveillance strengthening activities in response to the COVID-19 pandemic while simultaneously developing a vision to support countries in sustainably integrating this work into broader public health systems. The regional policy framework is intended to foster greater harmonization across the region’s genomic surveillance activities and aims to take a holistic approach to building capacities across regional flagship priorities. A regional strategy endorsed by the WHO Regional Committee for South-East Asia in 2022 is guiding these efforts: the South-East Asia Regional Roadmap for Diagnostic Preparedness, Integrated Laboratory Networking and Genomic Surveillance (2023–2027) (18). A key element of the regional strategy is the establishment of a regional genomic surveillance consortium. The consortium is envisioned as a country-led mechanism to decentralize genomic surveillance capacities to the national and subnational levels in the region through multisectoral partnership and collaboration while also building a trustworthy architecture for the rapid sharing of information for risk assessment and public health decision-making. The strategy also aims to strengthen national systems and collaborative efforts of epidemiologists and sequencing laboratories in the region to generate improved strategic information, including through epidemiological and laboratory investigations.

### Western Pacific region

The WHO Western Pacific Regional Office launched the Western Pacific Region Emerging Molecular Pathogen Characterization Technologies (EMPaCT) Surveillance Network in 2021 to support the creation and strengthening of sustainable in-country genomic surveillance systems (19). EMPaCT is aligned with the Asia Pacific Strategy for Emerging Diseases and Public Health Emergencies (APSED III), which guides implementation of the International Health Regulations (2005) (20). EMPaCT encourages countries in the region to work toward achievable goals in three phases. The first phase focuses on the detection, monitoring and characterization of known SARS-CoV-2 variants of concern (VOC) and variants of interest (VOI). The second phase expands the focus to the detection, monitoring and assessment of new VOC and VOI. The first two phases provide a foundation for the final phase: maintaining a comprehensive surveillance system that can detect, characterize and respond to emerging pathogens. Being able to assess the transmissibility, severity and impact of pathogens under investigation is emphasized as a core capacity for all countries in the region, regardless of their in-country genomic sequencing capability. The EMPaCT Network provides a platform for countries to discuss shared concerns about issues such as representative sampling and data sharing, while also facilitating coordination between countries and partners.

## Shared pathways to strengthening genomic surveillance across regions

Alongside considerable regional diversity, a number of common pathways to strengthening genomic surveillance for pandemic preparedness and response can be observed across all six WHO regions.

First, it is vital for national leaders and senior health officials to recognize the value of genomic surveillance, particularly during periods when political interest has waned. This requires raising awareness about how genomic data combined with additional sources of information in multisource health information systems can contribute to strategic decision-making, including during health emergencies. The Global Influenza Surveillance and Response System serves as an instructive example in this regard, and may provide opportunities to leverage existing resources to monitor disease trends and respond to outbreaks at the human-animal interface (21). Multistakeholder especially One Health advocacy initiatives are needed to build greater political support for genomic surveillance, with the dual objective of promoting the translation of genomic surveillance data into evidence-informed policies and encouraging sustained financial investment in these systems. Regional governance mechanisms provide important framing for countries on how to translate global strategic objectives into context-appropriate national policies, as demonstrated by the relevant regional strategies endorsed by the committees governing WHO’s Region of the Americas and South-East Asia Region (14, 18, 22).

Second, the development of strong monitoring and evaluation frameworks to accompany the implementation of national genomic surveillance strategies can help governments assess the benefits of investing in these systems. Determining how to measure the impact of genomic surveillance data on decision-making and on health outcomes is an important issue to be explored, as using effective indicators to demonstrate impact will strengthen the rationale for maintaining genomic surveillance capability. A global monitoring framework is being developed in line with the Global Strategy and reflects the lessons learnt from regional experiences.

Third, the differential toll of COVID-19 across and within countries has brought health equity to the forefront of the global dialogue about pandemic preparedness and response including under the aegis of a Pandemic Accord (23). The question of how to foster equitable access to genomic surveillance infrastructure and resources, including access to rapidly evolving technologies, must continue to be emphasized as countries work to develop their pandemic preparedness and response capabilities (24). A laboratory costing tool for genomic sequencing is being developed with partners to support decision-making and to articulate the needs. The tool builds on the WHO European Region’s “Better Labs for Better Health” initiative, which provides a framework for costing and implementing national laboratory service strengthening (25). This example highlights how global action has been driven by regional initiatives to bring good practices and tools to countries worldwide.

Fourth, given the challenges associated with establishing and sustaining national genomic sequencing capability, it is critical for countries without existing capability to have access to networks of reference laboratories that can provide timely sequencing results. These networks also can perform the vital function of analyzing and reporting on pooled genomic sequence data to identify new pathogens or variants that might not be detectable in country-level analyses of smaller numbers of sequences. Such networks can benefit from coordination with existing global disease surveillance networks with reference laboratories, such as those for influenza, measles/rubella, antimicrobial resistance, tuberculosis and HIV.

Fifth, stakeholder networks that were established or rapidly expanded in response to the COVID-19 pandemic have proven valuable for reducing duplicative efforts, disseminating best practices, and rapidly responding to new challenges and opportunities (26). Effective partner mapping and strategic partner engagement are key to further strengthening coordination and collaboration within and between these networks in the context of pandemic preparedness and response more broadly.

## Discussion

Countries in all regions increased their genomic sequencing capability during the COVID-19 pandemic. Historically, however, investment in pandemic preparedness and response has been characterized by cycles of “panic and neglect,” with resources often reduced during non-emergency phases to the extent that health systems are not ready to respond in a timely manner to new emergencies (27). In this context, strong measures are needed to sustain recent genomic surveillance achievements, reduce inequitable access to scientific technology, and strengthen our collective global health security by ensuring the use of geographically representative genomic surveillance data for better strategic decision-making. WHO’s six regional offices serve as platforms for countries in their respective regions to share knowledge and resources, engage stakeholders in ways that reflect national and regional priorities, and develop regionally harmonized approaches to sampling, data sharing, building the laboratory workforce, and other aspects of genomic surveillance.

Implementing the Global Strategy in regionally contextualized ways is key for country impact. For example, phased approaches have been suitable in the Region of the Americas and the Western Pacific Region, both of which have numerous small island developing states. These approaches take into consideration the varying degrees to which genomic surveillance has been implemented by different countries and provide pathways for countries to follow as they incrementally expand their genomic surveillance activities. The European Region and African Region, which together contain more than half of WHO’s 194 Member States, have benefitted from investment in subregional genomic surveillance capacity-strengthening hubs. In the South-East Asia Region, which includes highly populous countries such as Bangladesh, India and Indonesia, activities are underway to leverage regional assets by convening a consortium of policymakers, technical experts, donors, academic researchers and private-sector partners to strengthen and sustain genomic surveillance across all countries in the region. In the Eastern Mediterranean region, it is recognized that countries with complex emergencies require more robust external support for genomic surveillance than other countries. The regional approach flexibly addresses the needs of countries with complex emergencies while also strengthening the regional reference laboratories and facilitating strong linkages with international partners and networks.

In conclusion, cohesive global and regional approaches to supporting country-led efforts are required to realize the full potential of genomic surveillance as a pandemic preparedness and response tool. Ensuring that all countries have genomic sequencing capability or timely access to genomic sequencing for pathogens with pandemic and epidemic potential by 2032 is a shared endeavor, and challenges to implementing the Global Strategy must be addressed collaboratively at the national, regional and global levels. In the same way that pre-existing genomic surveillance capacities were leveraged to facilitate widespread sequencing of the novel SARS-CoV-2, genomic surveillance capacities developed in response to COVID-19 can be leveraged by public health systems to address other infectious diseases such as Rift Valley fever, Zika and Disease X (28). By capitalizing on the flexibility of genomic surveillance platforms, diverse stakeholders have the opportunity to transform many areas of public health surveillance while sustaining the infrastructure and skills required to rapidly scale up pathogen genomic surveillance in response to future public health emergencies.

## Data availability statement

The original contributions presented in the study are included in the article/Supplementary material, further inquiries can be directed to the corresponding author.

## Author contributions

OWA and GS conceptualized this article. OWA and KS-H developed the first draft of the manuscript following regional consultations. Authors from regional offices provided data on regional capabilities and information on regional actions to adapt the Global Genomic Surveillance Strategy for Pathogens with Pandemic and Epidemic Potential 2022–2032 to regional contexts for implementation. All authors participated in scoping the manuscript, elaborating different sections, reviewing drafts and approving the submitted version.

## Conflict of interest

The authors declare that the research was conducted in the absence of any commercial or financial relationships that could be construed as a potential conflict of interest.

## Publisher’s note

All claims expressed in this article are solely those of the authors and do not necessarily represent those of their affiliated organizations, or those of the publisher, the editors and the reviewers. Any product that may be evaluated in this article, or claim that may be made by its manufacturer, is not guaranteed or endorsed by the publisher.
