# Supplementary material for: Strengthening pathogen genomic surveillance for health emergencies: insights from the World Health Organization’s regional initiatives
Source: Front Public Health. 2023 Jun 9;11:1146730. doi: 10.3389/fpubh.2023.1146730 (PMC10289157; doi:10.3389/fpubh.2023.1146730)

Supplementary Material

Strengthening pathogen genomic surveillance for health emergencies: insights from the World Health Organization's regional initiatives

Oluwatosin Wuraola Akande*, Lisa L Carter, Abdinasir Abubakar, Rachel Achilla, Amal Barakat, Nicksy Gumede, Alina Guseinova, Francis Yesurajan Inbanathan, Masaya Kato, Etien Koua, Juliana Leite, Marco Marklewitz, Jairo Mendez-Rico, Chavely Monamele, Biran Musul, Karen Nahapetyan, Dhamari Naidoo, Rachel Ochola, Mehmet Ozel, Philomena Raftery, Andrea Vicari, Pushpa Ranjan Wijesinghe, Joanna Zwetyenga, Kelly Safreed-Harmon, Céline Barnadas, Mick Mulders, Dmitriy I. Pereyaslov, Jilian A. Sacks, Taylor Warren, Sébastien Cognat, Sylvie Briand, Gina Samaan

*** Correspondence:** Oluwatosin Wuraola Akande: akandeo@who.int

Supplementary Figure 1. Map showing the World Health Organization regions

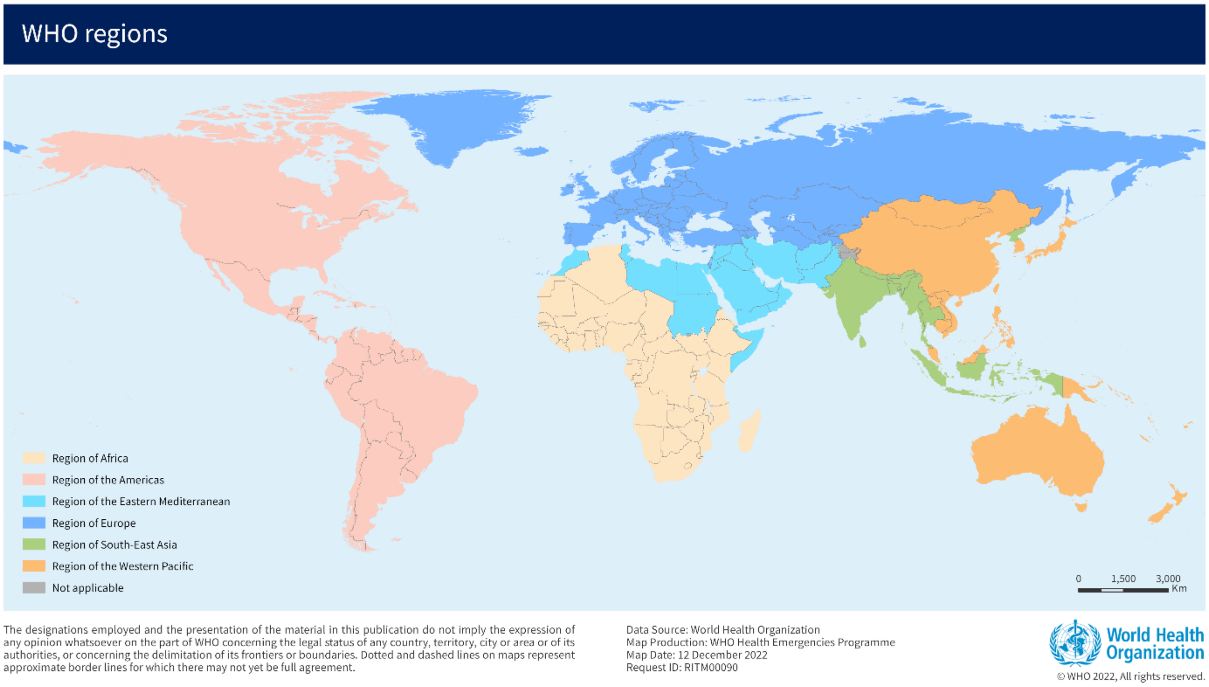

Supplement: Supplementary file 1 [file Image_1.pdf]
